# Evaluation of quality of life and description of the sociodemographic state in adolescent and young adult patients with phenylketonuria (PKU)

**DOI:** 10.1186/1477-7525-6-25

**Published:** 2008-03-26

**Authors:** Eva Simon, Martin Schwarz, Judith Roos, Nico Dragano, Max Geraedts, Johannes Siegrist, Gudrun Kamp, Udo Wendel

**Affiliations:** 1 Department of General Pediatrics, Heinrich Heine University, Duesseldorf, Germany; 2 Department of Medical Sociology, Heinrich Heine University, Duesseldorf, Germany; 3 Department of Gastroenterology, Hepatology and Infectious Diseases, Heinrich Heine University, Moorenstrasse, Duesseldorf, Germany

## Abstract

**Background:**

Normal intellectual and personal development can be expected in early-diagnosed and treated PKU patients. Aim of the study was to analyse quality of life and social status, which are important parameters for an overall estimation of success of treatment apart from intellectual outcome in adult PKU patients.

**Methods:**

67 patients completed a questionnaire on quality of life and social status. Data was compared to the German census on an age matched control collective.

**Results:**

Quality of life measured with the Profile of Quality of Life in the Chronically Ill (PLC) revealed mean values for capacity of performance in the patient group in the same range as in the control collective.

The analysis of the social state of PKU patients revealed a tendency towards lower or delayed autonomy, and a low rate of forming normal adult relationships in which to have children. Schooling and professional career corresponded approximately to the control collective.

**Conclusion:**

Though every chronic disorder must be regarded as restraining, it shows that PKU does not preclude healthy emotional adjustment when the disease is diagnosed early and treated well.

## Background

Phenylketonuria (PKU, McKusick 261600) is the most common error of the amino acid metabolism with an incidence of 1:12.000 in Germany. Due to a blockage in its degradation the essential amino acid phenylalanine accumulates resulting in severe mental retardation and neurological abnormalities. Treatment of PKU consists of a life-long protein-restricted diet with supplementation of phenylalanine-free amino acid mixtures. With the nation-wide introduction of newborn screening for PKU in Germany in the 1970s and the early institution of the diet, mental retardation due to PKU has been almost eliminated. Raised phenalanine levels in pregnancy exert adverse effects on the fetus (maternal PKU with microcephaly, growth retardation, developmental delay and congenital heart disease), therefore compliance with treatment must be especially strict.

Substantial knowledge has been gained of the intellectual [1], neurocognitive [2,3] and psychiatric state [4-7] of patients with phenylketonuria (PKU, McKusick 261600). This experience allows for a vast overview but further parameters are necessary for an overall estimation of a patient's outcome. Such important aspects of interest for the outcome of PKU patients are quality of life and social outcome.

Quality of life (QoL) is a multidimensional concept reflecting the impact of disease and treatment on a patient's subjective evaluation of his or her functioning and emotional well-being. PKU is a relatively benign disease with a good subjective physical function and a low number of hospitalisations. A completely normal personal development can be expected in early treated patients [8]. All the same, frequent blood tests for monitoring, the necessity of compliance with a complex diet, the occurrence of neurological symptoms and the stigma of the diagnosis of an inborn error of metabolism are features that make it reasonable to assume PKU may have impact on the quality of life in affected individuals.

In addition to a poor subjective perception of quality of life, suffering from a chronic inborn disease might impose special problems resulting in an exceptional social structure (interpersonal relationships, education and professional career) in comparison to healthy peers.

After the introduction of newborn screening for PKU in the 1960s, the number of early diagnosed and early treated adult patients continues to increase. Normal outcome is expected in these patients. High quality of life, a normal social status and an independent adult lifestyle are major goals of treatment.

## Methods

104 early treated PKU patients aged 17 years and above, who formerly or actually attended the metabolic units of the University Hospitals in Duesseldorf or Cologne, were invited by mail to participate in the study. A total of 67 patients completed and returned the standardized self-assessed questionnaire (response rate 64%). Time of questionnaire dispatch was July 2003.

Quality of life was enquired with the Profile of Quality of Life in the Chronically Ill (PLC) questionnaire [9]. The PLC is an approved questionnaire with a satisfactory criterion validity that has been used in a number of studies on QoL in chronic diseases. Data on the German population between 14 and 92 years have been collected for comparison of patient groups with a normal collective. The core module of this approved questionnaire is composed of 40 Likert-scaled items (scale 0–4) with 0 representing minimum and 4 representing maximum satisfaction. The items measure physical, psychological and social capacity of performance and well-being (for details see table 1). In addition to the core module, disease-specific symptoms depending on the investigated patient collective can be added to the questionnaire. Patients were asked if they suffered from memory impairment, headache, tremor of hands, tremor of arms and legs, lack of dexterity, slow reaction and skin abnormalities which are rather frequent symptoms in PKU [10-13] during the last seven days before filling in the questionnaire.

**Table 1:** Theoretical dimensions and factorial structure of the PLC (Siegrist et al 1996)

	Capacity of Performance	Well-being
Physical	I. Physical functioning	Listing of Symptoms
	(Performance capacity)	
	8 items	
Psychological	II. Psychological Functioning	III. Positive Mood (5 items)
	(Capacity of enjoying and relaxing)	IV. Negative Mood (8 items)
	(8 Items)	
Social	V. Social Functioning	VI. Social Well-being
	(Capacity of performing in social roles)	(Feelings of belonging)
	(6 items)	(5 items)

The QoL patient data were compared to data on an approximately age-matched control collective (aged 18–34 years) representative for the German population [14]. The patient collective was then divided into two age groups (≤25 years *vs. *>25 years) in order to detect possible age-dependent differences in QoL. Furthermore QoL in male and female patients was compared.

Data were analysed with SPSS-12 (SPSS Inc, Chicago, IL). The Wilcoxon Rank-Sum (Mann-Whitney-U), a non-parametric test (distribution-free) was used to compare differences. P < 0.05 was considered to indicate statistically significant difference. In case of multiple testing (e.g. quality of life subscales) p-values were rated using Bonferroni correction.

In the second section of the questionnaire, information about the patients' socioeconomic and sociodemographic status (marital status, children, type of habitation, school education, professional career, labour force) were collected. Frequencies were run and data were compared to data drawn from the German 2003 census on the age-matched general population of the same German province (North Rhine-Westphalia) where the patients lived and were treated for their PKU. As no data on type of habitation were available from the census of North Rhine-Westphalia, the German population (age 18–34 years) was taken as a control group for this parameter.

At the time of the survey the patients were aged between 17 and 38 years with a median age of 25 years. The median age was higher in the male than in the female patient collective (28,5 *vs. *23 years). Supposing an even age distribution, the median age in the general population would be 27,5 years.

The gender distribution in the patient group was uneven with 34% male and 66% female patients, therefore data were analysed separately for the male and the female patient groups.

No identifiers were present on the sent documents and the analysis was completely anonymous. The institutional review board of the Heinrich-Heine University fully approved the protocol for this investigation.

## Results

### Quality of life

No significant differences were detected between the self-assessed QoL in the patient group and the control collective. The mean values for capacity of performance and well being amount to 2,7–3,2 in the patient group and to 2,5–3,0 in the control collective (table 2). The mean number of disease-specific symptoms in the patient collective was 1 ± 0,9. Frequent symptoms were headache and poor memory.

**Table 2:** Comparison of mean QoL measured with the Profile of Quality of Life in the Chronically Ill (PLC) in PKU patients with the German norm; Mean and Standard Deviation

	Patient collective 17–38 years Mean (SD)	Normal collective 18–34 years Mean (SD)	Significance
Physical functioning	2.9 (0.6)	2.8 (0.6)	n.s.
Psychological Functioning	2.8 (0.7)	2.7 (0.6)	n.s.
Positive Mood	2.7 (0.8)	2.5 (0.7)	n.s.
Negative Mood	3.2 (0.6)	3.0 (0.8)	n.s.
Social Functioning	2.9 (0.9)	2.7 (0.7)	n.s.
Social Well-being	3.1 (0.7)	3.0 (0.7)	n.s.
Number of symptoms	1.0 (0.9)	-	

Patients older than 25 years stated more PKU-specific symptoms than younger patients (Table 3) but the difference did not reach statistical significance (1.2 *vs. *0.8, p = 0.08).

**Table 3:** Comparison of mean QoL in patients under and above 25 years; Mean and Standard Deviation

	Patient collective 17–25 years Mean (SD)	Patient collective 25–37 years Mean (SD)	Significance
Physical functioning	3.0 (0.8)	3.0 (0.4)	n.s.
Psychological Functioning	2.8 (0.8)	2.8 (0.5)	n.s.
Positive Mood	2.6 (0.9)	2.8 (0.6)	n.s.
Negative Mood	3.1 (0.6)	3.3 (0.4)	n.s.
Social Functioning	3.0 (0.7)	2.8 (0.7)	n.s.
Social Well-being	3.0 (0.8)	3.1 (0.7)	n.s.
Number of symptoms	0.8 (0.9)	1.2 (1.0)	n.s.

In the separate analysis of QoL of male and female patients (table 4) women stated lower levels of positive mood (Score 2.5 *vs. *3.0, p = 0.02) and lower levels of psychological functioning (Score 2.7 *vs. *3.0, p = 0.09). Women indicated higher scores in the category "social wellbeing" (Score 3.2 *vs. *2.9, p = 0.09). However, differences were small and p-values are above the defined threshold (p > 0.001; Bonferroni corrected)

**Table 4:** Comparison of mean QoL in male and female patients; Mean and Standard Deviation

	Patient collective Female Mean (SD)	Patient collective Male Mean (SD)	Significance
Physical functioning	2.9 (0.7)	3.0 (0.5)	n.s.
Psychological Functioning	2.7 (0.7)	3.0 (0.6)	(p = 0.09)
Positive Mood	2.5 (0.8)	3.0 (0.6)	(p = 0.02)
Negative Mood	3.1 (0.6)	3.3 (0.5)	n.s.
Social Functioning	2.9 (0.7)	2.9 (0.7)	n.s.
Social Well-being	3.2 (0.7)	2.9 (0.7)	(p = 0.09)

Range of scales: 0–4; low values = low quality of life, high value = high quality of life, SD, standard deviation; n.s., not significant

### Sociodemographic and socioeconomic data

The data on marital status, children and habitation are shown in table 5. Data on school and professional education are summarised in table 6.

**Table 5:** Sociodemographic data: Marital state, children, habitation

	Both sexes		Male		Female	
	Patient collective	Normal collective	Patient collective	Normal collective	Patient collective	Normal collective

Marital state						
Unmarried	82%	55%	83%	61%	82%	49%
Steady relationship	33%	n.a.	22%	n.a.	39%	n.a.
No steady relationship	67%	n.a.	78%	n.a.	61%	n.a.
Married	15%	42%	13%	37%	16%	47%
Divorced	3%	3%	4%	3%	2%	4%
Children	12%	47%	18%	33%	9%	51%
Type of habitation						
Single household	18%	23,1%	30%	27%	11%	20%
Parents	46%	24,6%	48%	26%	46%	24%
Non-relative (shared flat)	3%	n.a.	4%	n.a.	2%	n.a.
Marital or a illegitimate partner	31%	n.a.	17%	n.a.	39%	n.a.
Others	2%	n.a.	0%	n.a.	2%	n.a.

n.a., data not available from the German census

**Table 6:** School education and professional training

	Both sexes		Male		Female	
	Patient collective	Normal collective	Patient collective	Normal collective	Patient collective	Normal collective

Highest school leaving certificate						
Secondary school leaving certificate	22%	26%	35%	30%	16%	23%
Intermediate school leaving certificate	29%	26%	13%	23%	36%	29%
Entrance qualification for university	33%	33%	39%	33%	30%	34%
Others/no information	16%	14%	13%	14%	18%	14%
						
Persons still in professional training	43%	24%	35%	25%	48%	23%
Of these:						
School of general education	35%	26%	25%	24%	38%	28%
Apprenticeship	35%	49%	25%	50%	38%	47%
University student	29%	24%	50%	24%	19%	24%
Others/no information	3%	1%	-	-	5%	-
						
Highest professional qualification						
No professional training finished	48%	36%	25%	35%	55%	37%
Apprenticeship	43%	44%	52%	43%	39%	45%
Master craftsman/technician	2%	5%	-	6%	2%	4%
University degree	8%	9%	13%	10%	5%	9%
Others/no information	-	5%	-	6%	-	5%
						
Labour force	73%	75%	74%	84%	73%	66%
Of these:						
Unemployed	8%	11%	6%	12%	9%	9%
Part-time employed	22%	18%	24%	6%	22%	32%
Full-time employed	69%	72%	71%	82%	69%	59%

A great percentage of patients still lived with their parents (48% of the male and 46% of the female patients) in contrast to approximately one quarter of the general population.

The analysis of marital state revealed a higher percentage of patients being unmarried in comparison to the general population. This applied for the complete patient group as well as for the male and female group taken separately (>80% *vs. *50–60%). The main proportion of the unmarried patients were not in a steady relationship, in the male patients this ratio was as high as 95%. While nearly half of the subjects in the general population had children, this was the case in only approximately 9% of the female and 18% of the male adult patients.

Concerning school education no obvious differences were detected between patients and the general population. The distribution of the highest professional qualifications was the same, the only remarkable feature was that more than half of the female patients had not finished a vocational training at the time of the inquiry in contrast to approximately one third in the general population.

The labour force status (table 6) in the patient collective resembled the status in the general population. Differences were a higher percentage of part-time employees in the male patient group than in the male general population, while the proportion of part time employees was slightly lower in the female patient group than in the normal population.

## Discussion

### Quality of life

QoL is a multidimensional measure that is increasingly being used to evaluate outcome apart from clearly verifiable clinical symptoms. Physical, emotional, and social factors of subjective well being are summarised in this measure. QoL is suitable for the evaluation of affection by illness and treatment, and therefore might help illuminate areas of concern to a patient that have been previously under-recognised.

Till date, few studies investigated QoL as an outcome measure in PKU patients. In different studies on psychopathology in PKU in the 1990s, adolescent PKU patients stated reduced self-autonomy and a restricted social situation [15-19]. The necessity of compliance with a diet created a feeling of being poorly integrable in their peer groups [20]. In 37 Swiss patients between 3 and 18 years a normal QoL was stated in a standardised questionnaire filled in by the parents of the affected children. The only statistically significant abnormality in comparison to a healthy control collective was the presence of less positive emotions in the patient group [21]. In a recent study with 32 young adult Dutch patients self-assessed health-related quality of life was comparable to quality of life of controls [22].

In the present study QoL was measured with the Profile of Quality of Life in the Chronically Ill (PLC). In this questionnaire the two aspects of QoL, performance capacity and well being, are weighted equally resulting in an evaluation of negative feelings as well as constrictions in everyday life. The questionnaire has been used in different groups of chronically ill [9] and has been proven to have a satisfactory discriminant and criterion validity.

No significant differences were detected between the self-assessed QoL in adolescent and adult PKU patients and the control collective. Adults above 25 years stated more PKU specific neurological symptoms than younger patients but this difference did not reach statistical significance. Apart from a deterioration of neurological symptoms with advancing age as described in adult patients after the discontinuation of a diet [23,24], a possible cause might be a more intense reflection on the disease with advancing age resulting in the perception of more intense symptoms.

More intense reflection might also be the cause for a tendency towards reduced capacity of enjoying and relaxing and a less positive mood in the female patients. Additionally, to deal with the problem of maternal PKU might also result in a less positive mood in the female patient group.

The subjective appraisal of QoL might have been very positive because adult PKU patients possibly inadvertently choose the form of dealing with the disease resulting in the best subjective quality of life: Compliant patients are accustomed to the diet, so that they do not experience it as a restriction; in non-compliant patients the burden of the diet is inexistent and QoL is unimpaired as the subjects experience no acute clinical symptoms of PKU or are used to the mild symptoms.

Despite a normal estimation of QoL in the PLC, it is undeniable that PKU places a burden on a high number of patients. Compliance with the diet, frequent visits at the hospital and not least the sheer knowledge of the diagnosis of PKU are obvious stressors. Every chronic disorder must be regarded as restraining, however, it shows that PKU does not preclude healthy emotional and social adjustment when the disease is diagnosed early and treated well.

### Sociodemographic and socioeconomic data

Social outcome is an important measure for the success of treatment of an inborn chronic disease. In the present analysis a number of differences became manifest in the comparison between social outcome in a large collective of young adult classic PKU patients and an age-matched control collective.

In the patient group the marriage rate was low and only a very small number of unmarried patients were in steady relationships. Only 9% of the female and 18% of the male patients had children. A low number of stable relationships within to have children has been described frequently in patients with chronic diseases [24-27] and was attributed to poor body- or self-image. In contrast, a normal autonomy development, psychosocial development and social development was found in 32 adult Dutch PKU patients [22].

The analysis of type of habitation revealed an exceptionally high percentage of patients still living with their parents. This might be due to low autonomy in adolescent and young adult PKU patients in comparison to healthy members of the same age group. Suffering from a chronic metabolic disease with the necessity of parental control of behaviour and diet often results in overprotection in childhood with a delay in achieving autonomy [27].

A normal intellectual outcome can be expected in early-treated PKU patients. However, school performance as well as professional career does not only depend on intelligence. Recidiving elevations of phenylalanine concentrations creating transient neuropsychological deficits (impaired attention and short term memory) [2,3] as well as behavioural and emotional factors experienced by children and adolescents with a chronic disease may also play a role for academic achievement. Previous studies evaluating school careers in patients with PKU came to different results: German, Swiss and French studies did not detect differences in school career comparing affected subjects to the general population. In contrast, such differences were present in a considerable part of American, British, Polish, Hungarian, Austrian, Czech and Spanish PKU patients [28-30]. A recent Dutch study revealed that a high percentage of PKU patients needed special education in primary school, yet the final educational performance of the patients was normal [22]. In our study collective the distribution of the highest school leaving certificates in the patient group differed discretely from the frequencies in the normal collective without a clear trend towards lower education in the patients. Concerning professional career, the high rate of female patients who had no professional qualification might be due to their younger age in comparison to the control collective, but might as well be a sign of delayed psychosocial development with unusually long dependency on the parents without seeking economic and social independence.

The observed higher percentage of part-time employment in male patients might be a reflection of a lower achievement potential in chronically ill patients [26].

Taken together there is a tendency towards lower or delayed autonomy possibly due to overprotection and a low rate of forming normal adult relationships in PKU patients.

## Conclusion

The analysis of the social state of PKU patients revealed a tendency towards lower or delayed autonomy, and a low rate of forming normal adult relationships in which to have children. Schooling and professional career corresponded approximately to the control collective. Quality of life measured with the Profile of Quality of Life in the Chronically Ill (PLC) revealed mean values for capacity of performance in the patient group in the same range as in the control collective. Though every chronic disorder must be regarded as restraining, it shows that PKU does not preclude healthy emotional adjustment when the disease is diagnosed early and treated well.

Limitations of the present study should be acknowledged: Although the recruitment rate was high, the sample size was still small. It has to be assumed, that track of a considerable percentage of patients was lost in late childhood and adolescence. Thus, it is unclear to what extent our results can be transferred to the overall PKU population. Selection bias with the inclusion of only compliant, socially adjusted patients has to be considered.

Furthermore, the small size of the sample limits the power to detect subtle effects. Areas of concern to the patient might have been under-recognised with the use of standardised questionnaires with formalised questions on categories chosen by the investigators.

## Abbreviations

PKU: Phenylketonuria; QoL: Quality of life; PLC: Profile of Quality of Life in the Chronically Ill.

## Competing interests

The author(s) declare that they have no competing interests.

## Authors' contributions

ES did the data analysis and drafted the manuscript. MS and UW conceived of the study and designed the questionnaires. Both conducted the realisation and the analysis of the study and helped to draft the manuscript. JR participated in the design of the study and participated in the analysis of the questionnaires. JR did the data collection. ND, MG and JS participated in the design and the data analysis. ND did the statistical analysis. GK participated in the design of the questionnaires and helped to draft the manuscript. All authors read and approved the final manuscript.
